# The contribution of Latin American research to HPV epidemiology and natural history knowledge

**DOI:** 10.1590/1414-431X20199560

**Published:** 2020-01-31

**Authors:** L. Sichero, M.A. Picconi, L.L. Villa

**Affiliations:** 1 Centro de Investigação Translacional em Oncologia, Instituto do Câncer do Estado de São Paulo, Hospital das Clinicas, Faculdade de Medicina, Universidade de São Paulo, São Paulo, SP, Brasil; 2 Oncogenic Viruses Service, National and Regional HPV Reference Laboratory, National Institute of Infectious Diseases - ANLIS “Dr. Carlos G. Malbrán”, Buenos Aires, Argentina; 3 Departamento de Radiologia e Oncologia, Faculdade de Medicina, Universidade de São Paulo, São Paulo, SP, Brasil

**Keywords:** HPV, Latin America, Immunization, Cervical cancer, Natural history, Screening

## Abstract

Our aim was to review the major contributions of studies conducted in different Latin American (LA) countries to the field of human papillomavirus (HPV) epidemiology, natural history, risk of disease, and prevention strategies, mainly in the uterine cervix. Although cytological screening is established in several countries in LA, incidence and mortality rates from cervical cancer (CC) are still extremely high. Finally, data from large cohort studies conducted in LA countries provided seminal data to propose primary and secondary prevention modalities: the HPV vaccine has been introduced in the national immunization programs of several LA countries and multiple screening experiences using HPV testing are under evaluation in the region.

## Introduction

Human papillomaviruses (HPVs) are a diverse group of epitheliotropic viruses that may cause warty and neoplastic changes in the epithelia at different anatomical sites. HPVs consist of a non-enveloped virus with a 50-nm diameter capsid that encloses a single circular double stranded DNA molecule of approximately 8,000 bp associated to cellular histones. The viral genome encodes six early (E1, E2, E4, E5, E6, and E7) and two late proteins (L1 and L2). While both late proteins associate for capsid formation, early proteins control viral transcription and replication, and are involved in cellular transformation (1).

HPV DNA integration, mostly observed in malignant cervical lesions, preferentially occurs within transcriptionally active and enhancer regions of the cell genome (1). The consequence of viral DNA disruption is constitutive E6 and E7 expression followed by selective growth advantage as compared to cells with only epissomal viral DNA. High-risk HPVs E6 and E7 proteins interact with and suppress the function of TP53 and pRB proteins, respectively, both master regulators of the cell cycle, thus extending the life span of infected cells favoring the accumulation of mitotic defects, genomic instability, and ultimately neoplasia development.

HPV taxonomy and phylogeny relies on the complete L1 nucleotide sequence; it is established that viral types differ by at least 10% within this genome region. For this reason, distinct from most other viruses, HPV detection and genotyping is evaluated via DNA analysis instead of viral isolation. To date, over 200 HPV types are completely sequenced and characterized, and numbered in the order of their discovery (2). Among these, about 40 can infect the human mucosa, particularly the anogenital and aerodigestive tracts, albeit most of our understanding concentrates on cervical HPV infections. The International Agency for Research on Cancer (IARC) has classified 13 HPV types as group 1 carcinogens (HPV-16, -18, -31, -33, -35, -39, -45, -51, -52, -56, -58, -59, and -66), which are commonly referred to as “high risk-HPVs” (hr-HPVs) (1). Among these, hr-HPV-16 is undoubtedly the most carcinogenic based in the burden of cervical cancer (CC) and its precursor lesions. hr-HPV-16 further responds for most HPV-related cancers in other anogenital areas and the oropharynx (1). Although hr-HPV-18 is a distant second in terms of etiologic importance, this viral type accounts for a high fraction of adenocarcinomas. On the other hand, infections by non-oncogenic or low-risk HPV types (lr-HPVs) -6, -11, -40, -42, -43, -44, and -54 are epidemiologically associated to benign proliferations such as condylomas and genital warts.

Sequence comparison of worldwide collected isolates from the same HPV has revealed some intratypic nucleotide heterogeneity. Accordingly, the International Papillomavirus Nomenclature Committee has designated that HPV genomes are classified into molecular variants of the same HPV when these present over 98% of identity within the L1 gene sequence (3). Viral variants mostly arise by nucleotide substitutions in a few restricted positions within the entire genome. Due to its global predominance and clinical importance, by far the most extensive worldwide studies concerning HPV intratypic nucleotide heterogeneity have been conducted for hr-HPV-16, followed by hr-HPV-18 (3,4), and during the last decade for other viral types considered type 1 carcinogens (5). Altogether, these studies were crucial to elucidate different features of HPV phylogenetic relationships. For instance, at least for hr-HPV-16 and -18, it is estimated that the nucleotide diversity observed represents over 200,000 years of evolution from a precursor genome probably originated in Africa, and that was further segregated in phylogenetic related lineages: European (E), Asian-American (AA), Asian (As), and African (Af) (9). The worldwide circulation of the different HPV-16 molecular variants varies significantly and correlates with the intrinsic admixture level of each population (3,4).

## Epidemiology of HPV-related neoplasias in LA

Since the early 1970s, a link between HPV infection and the development of neoplastic lesions was observed. Infections by these viruses are strongly associated to the development of cancer of the cervix, vagina, and vulva in women, penis in men, and anal canal and head and neck (mostly in the oropharynx) cancers in both genders. Additionally, genital warts and the rare but very serious recurrent respiratory papillomatosis are etiologically associated with HPV infection (1). The importance of these findings is highlighted by the 2008 Nobel Prize to Prof. Harald zur Hausen for his discovery that hr-HPVs are the causative agents of CC.

Cervical cancer is the third most frequent cancer among women worldwide. Importantly, CC remains a significant problem mainly in underdeveloped and developing countries where approximately 80% of the cases occur. Latin America (LA) bears one of the highest incidence rates of CC with age-adjusted incidence rates ranging from 10 to 80 per 100,000 women/year (6). Nevertheless, CC rates vary considerably throughout LA, ranging from 11.4 cases per 100,000 women/year in Costa Rica to 47.7 cases per 100,000 women/year in Bolivia.

Decades of Papanicolaou-based screening to detect pre-cancerous cervical lesions in a few countries of the region have not had a major impact in reducing CC incidence and mortality rates, which are still high across LA. Moreover, estimates indicate an overall 72% increase in the incidence of CC and a 78% rise in mortality by this disease between 2012 and 2030 (6). Each year, about 1.1 million new CC cases and 600,000 deaths are recognized in LA and the Caribbean. It is noteworthy that even though breast cancer remains the global leading cause of women death, CC surpasses this neoplasia in Bolivia, Honduras, and Nicaragua. Barriers to control CC in LA include uneven allocation of resources, variable infrastructure and service availability, limited number of population-based cancer registries, and scarce distribution of public health centers, which is even more evident in rural areas far from the large urban centers. Taken together, these difficulties result in a scenario of unequal care provided to cancer-affected individuals.

The global burden of HPV infections and associated diseases is significant. Particularly within LA, HPV was associated to 83,195 new cases of CC and 35,673 associated CC deaths in 2012 (7). Most cases are associated to hr-HPV-16 and -18, followed by hr-HPV-31, -33, -45, -52, and -58, which together account for approximately 90% of CC cases worldwide. Nevertheless, in LA countries information remains scarce concerning HPV-related tumors aside from the uterine cervix. A recent systematic review regarding the prevalence of HPV in non-cervical anatomical regions revealed not only high HPV prevalence at these sites but also higher clearance rates of infection compared to the uterine cervix (8). In the northeast region of Brazil and some areas of Argentina, anal canal cancer incidence rates reported are 0.2 to 1.4 per 100,000 persons/year (6); for other countries in LA, data are still very limited or even non-existent concerning anal canal cancer prevalence. This neoplasia is associated to hr-HPVs, particularly hr-HPV-16.

Vulvar and vaginal cancers are relatively rare tumors with worldwide incidence rates below 1 per 100,000 women/year. Very scarce information concerning cancer at these anatomical sites is available in LA countries. Nevertheless, regional data shows that 75-100% of the basaloid/warty vulvar cancers, which are more common in young women, are hr-HPV-16 positive (7).

Penile cancer is more commonly detected in men aged 50–70 years, with a worldwide incidence of 34,475 cases per year. Although very rare, penile cancer incidence rates are higher in less-developed countries, including some countries in LA, and responds for up to 10% of male tumors in some parts of Africa, Asia, and South America. In Brazil, this neoplasia accounts for about 2% of all male cancers, and both the North and Northeast regions are the most affected. Furthermore, within the central region of Brazil and some areas in Colombia and Paraguay, incidence rates are about 2.0 per 100,000 men/year, much higher compared to other countries where rates reported are around 0.4 per 100,000 men/year (6). Studies performed in some countries in LA show HPV DNA positivity among 30–50% of penile cancers (8).

Within the head and neck, cancers at specific anatomical sites are associated to HPV infection with variable frequencies; HPV-associated cancers are mostly observed in the base of tongue and tonsils (9). Nevertheless, worldwide reported prevalence rates of HPV DNA in head and neck squamous cell carcinoma (HNSCC) vary considerably, ranging from 0 to 100% in the oropharynx. Hr-HPV-16 is by far the most prevalent viral type not only globally, but also in different tumor series analyzed in LA countries (10). It is important to highlight that several reports revealed lower HPV positivity in oropharyngeal cancers from LA countries compared to other developed countries in the Northern hemisphere particularly the USA, Sweden, and Australia, where it causes over 50% of the cases (11 [Bibr B12] [Bibr B13] [Bibr B14] [Bibr B15] [Bibr B16] [Bibr B17] [Bibr B18] [Bibr B19] [Bibr B20] [Bibr B21] [Bibr B22] [Bibr B23] [Bibr B24] [Bibr B25]). Further studies are necessary to better comprehend the basis for such differences and the impact on cancer patient management.

## Natural history of HPV infection and research in LA

HPV-induced cervical carcinogenesis is the most comprehensively studied neoplasia associated to HPV infection. CC results from distinct and sequential steps including infection of the cervical transformation zone metaplastic epithelium with one or more hr-HPV types, viral persistence, clonal progression of the persistently-infected epithelium to cervical precancer, and eventually invasion (1). Several epidemiologic studies conducted in LA countries have significantly contributed to better understand these fundamental stages of cervical carcinogenesis and to identify factors affecting each the transitions. Supplementary Table S1 summarizes the main findings of the most relevant studies conducted in LA, among which we should highlight the importance of not only case-control studies conducted by the IARC in Colombia, Paraguay, Brazil, and Peru, but also longitudinal cohort studies of Proyecto Guanacaste (Costa Rica) and the Ludwig-McGill cohort (Brazil), and the HPV prevalence studies by the IARC in Colombia, Mexico, Argentina, and Chile.

HPV infection is considered the most commonly diagnosed sexually transmitted disease. The vast majority of individuals who engage in sexual activity will become infected with HPV at least once during their lifetime (1). Anogenital HPV infections are primarily transmitted by sexual contact in both genders, and it is believed that HPV infections are easily transmitted through microscopic traumas in the mucosa or skin resulting from sexual intercourse. Consequently, HPV prevalence peaks around the sexual debut age when exposure is high, and markers of sexual activity including age at first sexual intercourse and high number of recent/lifetime sexual partners are important risk factors for HPV infections. In addition to peno-vaginal contact, HPV is also transmitted by other sexual practices, including peno-anal intercourse, oral sex, and digital-vaginal sex. While it was observed that the risk of hr-HPVs infection is associated with sexual variables, the risk of lr-HPVs infection seems to be predominantly associated with sociodemographic- and hygiene-related variables.

Most HPV infections are transient and viral clearance by the immune system occurs spontaneously without ever causing lesions. In fact, infections become undetectable within 2 years in more than 90% of affected individuals (1). Nevertheless, a small proportion of HPV infections, particularly with hr-HPV types, may take longer to clear. Hr-HPV infections that “persist” are more likely to progress to true CC precursor lesions, i.e., high-grade squamous intraepithelial lesions (HSIL) or cervical intra-epithelial neoplasia grade 3 (CIN3), and evolution to CC may take several years if left untreated. Persistent infection by hr-HPVs is responsible for the development of the majority, if not all, CC worldwide.

Cervical HPV infection is age-dependent and an inverse relationship between age and HPV prevalence has been reported. HPV prevalence peaks below age 25 shortly after sexual initiation for most women, and declines with age. Data from the Guanacaste cohort (26) and the TATI project (53 [Bibr B54] [Bibr B55] [Bibr B56] [Bibr B57] [Bibr B58] [Bibr B59] [Bibr B60] [Bibr B61]) show an overall HPV prevalence of 26% in women younger than 25, falling to 12% in those aged 35–44, however, rising again to 22% in women older than 54 years. This U-shaped age-specific curve of hr-HPVs prevalence corroborates data from independent studies conducted in Costa Rica (27 [Bibr B28] [Bibr B29] [Bibr B30] [Bibr B31] [Bibr B32]), Mexico (39), Chile (41 [Bibr B42] [Bibr B43] [Bibr B44] [Bibr B45] [Bibr B46] [Bibr B47] [Bibr B48] [Bibr B49] [Bibr B50] [Bibr B51] [Bibr B52]), Brazil (33 [Bibr B34] [Bibr B35] [Bibr B36] [Bibr B37]), and Colombia (38). The second peak of HPV infection among elder women could possibly be attributed to hormonal changes preceding menopause, alterations of male/female sexual behavior, or even higher rates of HPV persistence at older ages. Noteworthy, in Argentina, the curve peaked below age 25 and then dropped and plateaued around 30–35 years, reaching its minimum in women older than 65 (40), which more closely resembles the age-prevalence pattern of HPV infection reported in Europe and North America.

Viral persistence is more prevalent among women over 30 years, and infections with hr-HPVs have been shown to last longer than lr-HPVs infections (1). The relative risk of developing CC in patients with hr-HPV persistent infections is on average 50-fold higher compared to HPV-negative women. CIN occurs mostly in women between 25 and 35 years of age, whereas the incidence of CC takes place between 55 and 65 years, indicating a latency period of several years between initial infection and the development of CIN, and progression to invasive cancer.

Most HPV infections do not result in the development of cervical lesions and may be eliminated by the immune system in a short period of time. Nevertheless, the extent to which viral infections are cleared remains a major unresolved question. Approximately 60% of these infections will prompt type-specific seroconversion, and clearance of an HPV type leaves the individual partially immune to that genotype. Severe alterations in the immune system result in higher prevalence of clinically relevant HPV infections. In fact, co-infection with HIV has been shown to impair cell-mediated immune control of HPV infections (62). Host genetics and other influences on host immunity might also affect the immune response to HPV infection; weak associations of human leukocyte antigen DRB1 and DQB1 genes with risk of CIN3 have been described (63).

Even though hr-HPV DNA is detected in almost all CC cases, HPV infection alone is not sufficient to drive the full carcinogenesis process. The high prevalence of HPV infections in the female population and the relative low rate of CC indicate that both exogenous and inherent factors of the virus and the host can impact HPV natural history. A substantial part of the evidence of risk factors associated to HPV infection and lesion progression comes from studies conducted in LA. The lifetime number of male sexual partners and male partner sexual behavior significantly increase the risk, whereas circumcision is related to a reduction in the risk of HPV infection. Pre-existing HPV infection(s) is also associated with higher risk of acquiring another HPV type in addition to multiparity, long term use of oral contraceptives, and tobacco smoking. The role of chronic inflammation, especially from coinfection with *Chlamydia trachomatis* and certain dietary deficiencies have also been reported (64). Virus-related factors have also been implicated in HPV-induced carcinogenesis. For instance, for hr-HPV-16 (and other carcinogenic types) intratypic variants were shown to be relevant to the natural history of cervical neoplasia (65,66). Furthermore, viral loads were also clearly associated with concurrent disease detection and risk of neoplasia.

## HPV prevalence and type distribution in normal cytology and cervical lesions in LA

The analysis of HPV type prevalence and distribution in normal cytology and low grade squamous intra-epithelial lesion (LSIL) cervical samples reveals a wide spectrum of HPV types, both lr- and hr-HPVs, and as the severity of cervical lesions increases, hr-HPVs predominance increases, being the unique types detected in CC. Globally, hr-HPV-16 DNA is detected in 30% of cytologically normal HPV positive cervical samples and in 50–90% of HPV-positive CC. Additionally, hr-HPV-18 accounts for 10 to 20% of all CC cases. A wide variation in the prevalence of the lesser prevalent HPV types throughout the world is observed (67).

Regarding countries from LA, the prevalence of any HPV type observed was 16.1% in an extensive meta-analysis including 48,171 women with normal cytology from studies conducted in Trinidad & Tobago, Costa Rica, Honduras, Guatemala, Belize, Mexico, Argentina, Brazil, Chile, Colombia, Paraguay, and Peru. Specifically, the vaccine-targeted hr-HPV-16 and -18 were identified in 4.3% of normal samples. In LSIL samples from LA, the most common viral types identified were hr-HPV-16 (26%), hr-HPV-33 (13%), lr-HPV-6 (11%), hr-HPV-58 (8%), and hr-HPV-31 (7%). Finally, among 2,446 HSIL cases, 46.5 and 8.9% were shown to be hr-HPV-16 and -18 positive, respectively, while in 5,540 CC cases, 53.2% of cases harbored hr-HPV-16 and 13.2% hr-HPV-18. The following five most prevalent types, in decreasing frequency, were hr-HPV-31, -58, -33, -45, and -52 (68).

More recently, the worldwide meta-analysis regarding the cross-sectional viral type prevalence and distribution in HPV-positive women of all clinical status (from normal cytology to CC) accessed by PCR-based methods included 35,895 samples from South and Central America studies (67). Overall, HPV prevalence increased linearly with severity of cervical disease from 24% in normal cytology (substantially higher than worldwide prevalence estimates) to up to 90% in CC. As expected, hr-HPV-16 was the most frequently detected viral type across all cytological categories and varied slightly between normal cytology (16.1%) and LSIL (25.1%), but substantially increased in HSIL (53.5%) and CC (59.5%).

The prevalence of hr-HPV variants and their association with CC has been reported in three case-control studies (65,69,70), and five cross-sectional studies in LA women with different grades of cervical lesions (71–73). Taken together, these studies conducted in Argentina, Brazil, Costa Rica, Honduras, Mexico, and Paraguay have shown a broad diversity and distribution of hr-HPV-16 variants, with a higher prevalence of E variants, followed by variants from the Asian-American branch (Supplementary Table S2). Interestingly, a high prevalence of E variants was also observed in indigenous groups from Argentina (64,72,74). These studies further suggested that the colonization of the American continent by Europeans and Africans is reflected in the composition of its HPVs variants.

Several studies conducted in Mexico, Costa Rica, and Brazil have shown that non-E hr-HPV-16 variants, mostly AA variants, are associated with a higher risk of viral persistence and/or HSIL and CC development (65,69,70,75 [Bibr B76] [Bibr B77] [Bibr B78] [Bibr B79] [Bibr B80] [Bibr B81] [Bibr B82]). These studies that include a large number of samples provide enough power to detect associations between low prevalent variants and persistent infections or disease risk. It is noteworthy that *in vitro* functional assays have also shown that different hr-HPV-16 variants exhibit differences in their biological and biochemical properties, and further attributed to AA variants an increased oncogenic potential compared to E variants (3). Taken together, the data provided may explain the increased oncogenic potential reported for these variants and their contribution for the high incidence of CC.

## Primary and secondary HPV prevention: contribution of research in LA

HPV vaccines are based on virus-like particles (VLPs) composed of L1 protein, the main viral capsid constituent. Since 2006, three vaccines have been approved in LA for HPV prophylaxis: a bivalent vaccine composed of VLPs of hr-HPV-16 and -18 (GlaxoSmithKline, UK), a quadrivalent vaccine containing VLPs of hr-HPV-16 and -18 and lr-HPV-6 and -11 (Merck & Co., Inc., USA), and more recently a nine-valent vaccine containing VLPs for HPV types -6, -11, -16, -18, -31, -33, -45, -52, and -58. Large phase II and III clinical trials to evaluate prophylactic efficacy have been conducted in which both HPV infection and cervical disease endpoints were evaluated, particularly HSIL (CIN2 or CIN3) in addition to vulvar and vaginal intraepithelial neoplasias (VIN or VaIN, respectively), anal infection and dysplasia, and genital warts (83). Within different populations, including young women aged 16–26 and older women (up to 55 years), very high efficacy rates were observed. Additionally, the quadrivalent HPV vaccine has also proven to be efficacious in men for preventing genital and anal canal infections by viral types included in the vaccine and associated diseases. Importantly, several clinical trials regarding HPV prophylactic vaccines were conducted in LA countries, which together clearly demonstrated the safety, immunogenicity, and efficacy against different clinical endpoints of these recombinant vaccines among these populations (84). It is important to further highlight that the HPV vaccine acceptability is very high in the region.

In most LA countries, a well-developed public immunization infrastructure is available, which has facilitated the introduction of national immunization programs in these regions. Furthermore, the organizations United Nations Children's Emergency Fund (UNICEF), Global Alliance for Vaccines and Immunization (GAVI), and Pan American Health Organization (PAHO) Revolving Fund had a crucial role in enhancing HPV vaccine introduction in LA. Currently, 80% of girls aged 9–14 years in the LA have access to HPV vaccination through national immunization programs due to political will and the negotiated purchase of the vaccines. Moreover, in several countries in LA, the HPV vaccine is also accessible to HIV-positive women up to 26 years of age. In the last few years, HPV prophylactic immunization has been extended to boys and men living with HIV in several LA countries. The countries have adopted 2 doses (0–6 or 12 months) for children up to 14 years of age, and 3 doses (0–2–6 or 12 months) for all above that age. Nevertheless, significant challenges remain including the cost of the program, the variable coverage rates, and the monitoring of this intervention that requires tools and strategies unavailable in many countries of the region.

Since the introduction of HPV vaccination in national immunization programs, numerous articles on its impact in the real world have been published, supporting the effectiveness of an inclusive immunization program, preventing infection from spreading, and demonstrating potential for reducing cancer incidence in both genders (85). The impact and effectiveness of HPV vaccination seems to be higher when the vaccine is administered at a younger age, before sexual debut and exposure to HPV infection (86). Importantly, herd protection has been observed, prominently in heterosexual men of age groups close to the vaccinated subjects, but also in females in age groups close to that of the vaccinated subjects and dependent of high vaccine coverage. However, men who have sex with men and several high-risk groups, as immunosuppressed patients, are still being overlooked by the predominant HPV vaccination policies worldwide, perpetuating inequality in health care. Besides cost-related issues, LA countries may experience sociocultural, health systems, and political barriers to deliver and sustain HPV vaccination programs and opportunities to support these countries should be explored.

Recently, a broad evaluation of health programs is driving a revision of the entire CC control strategies adopted by each country, which comprises not only HPV vaccination of female adolescents but also cytology/HPV testing for adult women. Implementation with good coverage rates of cost-effective actions such as HPV vaccination alone or vaccination accompanied by screening will reduce HPV-related tumors in LA, as occurred in several countries of the world. For instance, in Argentina, a strong reduction in the prevalence of HPV 16/18 and closely related HPV types in sexually active adolescent women following the introduction of HPV vaccination was observed (unpublished data). Similar initiatives for the monitoring of HPV vaccine effectiveness are underway in Brazil. A nationwide HPV prevalence study of women and men from 16 to 25 years of age was recently finalized (87) to set the basis for the prevalence study among individuals after introduction of HPV vaccination in Brazil in 2014.

Cytology-based mass screening programs, which have been adopted as the standard care for CC screening for over 50 years, have been successful in reducing incidence and mortality in diverse developed countries. Unfortunately, most LA countries tried unsuccessfully to reproduce these results, evidencing however very high incidence and mortality rates, with no significant impact on the disease burden after decades of efforts (88). Inherent limitations in cervical cytology encouraged the development of new screening technologies including tests focusing on the detection of hr-HPV DNA. The advantages of these tests over cytology-based screening include greater sensitivity, high negative predictive value (which allows to extend the screening intervals in HPV-negative women), and automation. Nevertheless, the intrinsic problem of low positive predictive value of HPV testing once most HPV infections are transient and do not result in CC development is noteworthy. As a consequence, an additional test (“triage”) is required in women who are hr-HPV DNA positive in the primary screening in order to distinguish those who are at higher risk of having a CC from those harboring solely transient or lr-HPV infections. Triage includes visual inspection methods, cytology, and molecular biomarkers test (hr-HPV E6/E7 mRNA, hr-HPV E6 proteins, p16, among others). In fact, locally adapted algorithms employing primary screening with HPV testing are currently being developed in different settings.

Over the last decade, diverse experiences with HPV testing have been conducted in LA countries; some as part of research studies, others to pilot the implementation of HPV testing in the public health system, and more recently, the implementation of HPV tests as part of the public programs provided by the ministries of health (89). Pilot studies conducted in Argentina, Brazil, Chile, Colombia, El Salvador, Mexico, and Nicaragua were highly efficacious in detecting precancerous cervical lesions with good feasibility and acceptance of self-sampling. In fact, in 2011, Argentina was the first country in LA to implement HPV DNA testing for primary screening within the public health system for all women aged 30 or older. More recently, Mexico has expanded the implementation of HPV DNA testing to 17 sites across the country, and El Salvador, Guatemala, Honduras, and Nicaragua are beginning to institutionalize HPV testing at the population level (89). LA is slowly shifting towards HPV testing for CC screening, with the endorsement of several regional experiences that have resulted in increased coverage and better detection of precancer lesions using HPV tests. For instance, the ESTAMPA study, launched in LA countries by the IARC, will contribute with valuable information about the performance of emerging CC screening and triage techniques, and the feasibility of different approaches to implement organized HPV-based screening programs in the region (90).

## Future perspectives

Worldwide, hr-HPVs, primarily HPV-16, are the main etiological agents of almost all CCs and of an important fraction of tumors in the anogenital region and oropharynx of both women and men. Increasing knowledge concerning the activity of E6 and E7 viral oncoproteins revealed crucial molecular mechanisms that lead to cellular transformation. The prevalence of HPV infection at diverse anatomical sites and the HPV-related disease burden in different LA countries highlight the need of supporting CC prevention strategies in this region. CC is one of the leading causes of women deaths in LA and thus, requirement for CC prevention and control are inestimable.

The introduction of highly efficacious vaccines that prevent hr-HPV-16 and -18 infections (and other HPV types) revealed high potential of contributing to the reduction of a substantial proportion of tumors that without intervention would continue to increase in number. Although regional data point towards a favorable trend in prevention, important challenges remain, including the cost of vaccination programs, coverage of 2 vaccine doses, and the need to vaccinate susceptible populations like those living with HIV and other individuals with immunodeficiency. Equally important are the tools and strategies required to measure the impact of this intervention that are still unavailable in many countries of the region.

Considering secondary prevention strategies, most countries throughout LA attained no success in implementing cytology-based screening programs at the population level. However, after the introduction of prophylactic HPV vaccines, CC screening with HPV testing has proven to be more sensitive than Pap testing while allowing for an accurate monitoring of HPV-associated diseases. It is noteworthy that while screening is essential, it is only one component of many other aspects of population-based programs, which should be employed to effectively impact a reduction in CC cancer incidence and mortality. Challenges include the need to update screening guidelines, strengthen treatment capacity, and develop a comprehensive quality assurance plan for HPV testing.

Finally, several gaps still exist in the knowledge and the future lines of research, policy, and advocacy for non-cervical HPV cancer prevention, mainly regarding anal canal and oropharyngeal cancers and precursor lesions. Additional studies are necessary to better understand the natural history of viral infections, their pathogenesis and the impact on cancer patient management at these anatomical regions. Public health commitment and research to implement HPV-based preventive strategies, together with strong and common advocacy to overcome the barriers affecting the adoption of these strategies are likely to yield major benefits in reducing the burden of HPV-associated diseases in LA.

## Supplementary Material

Click here to view [pdf].
